# CPEB2-activated PDGFRα mRNA translation contributes to myofibroblast proliferation and pulmonary alveologenesis

**DOI:** 10.1186/s12929-020-00643-0

**Published:** 2020-04-15

**Authors:** Yen-Ting Lai, Hsu-Wen Chao, Alan Chuan-Ying Lai, Shu-Hui Lin, Ya-Jen Chang, Yi-Shuian Huang

**Affiliations:** 1 Institute of Biomedical Sciences, Academia Sinica, 128 Sec. 2, Academia Rd, Taipei, 11529 Taiwan; 2 Department of Physiology, School of Medicine, College of Medicine, Taipei Medical University, Taipei, 11031 Taiwan; 3 Graduate Institute of Medical Sciences, College of Medicine, Taipei Medical University, Taipei, 11031 Taiwan

**Keywords:** Alveologenesis, Bronchopulmonary dysplasia, CPEB2, Myofibroblast, PDGFRα, Translational control

## Abstract

**Background:**

Alveologenesis is the final stage of lung development to form air-exchanging units between alveoli and blood vessels. Genetic susceptibility or hyperoxic stress to perturb this complicated process can cause abnormal enlargement of alveoli and lead to bronchopulmonary dysplasia (BPD)-associated emphysema. Platelet-derived growth factor receptor α (PDGFRα) signaling is crucial for alveolar myofibroblast (MYF) proliferation and its deficiency is associated with risk of BPD, but posttranscriptional mechanisms regulating PDGFRα synthesis during lung development remain largely unexplored. Cytoplasmic polyadenylation element-binding protein 2 (CPEB2) is a sequence-specific RNA-binding protein and translational regulator. Because CPEB2-knockout (KO) mice showed emphysematous phenotypes, we investigated how CPEB2-controlled translation affects pulmonary development and function.

**Methods:**

Respiratory and pulmonary functions were measured by whole-body and invasive plethysmography. Histological staining and immunohistochemistry were used to analyze morphology, proliferation, apoptosis and cell densities from postnatal to adult lungs. Western blotting, RNA-immunoprecipitation, reporter assay, primary MYF culture and ectopic expression rescue were performed to demonstrate the role of CPEB2 in PDGFRα mRNA translation and MYF proliferation.

**Results:**

Adult CPEB2-KO mice showed emphysema-like dysfunction. The alveolar structure in CPEB2-deficient lungs appeared normal at birth but became simplified through the alveolar stage of lung development. In CPEB2-null mice, we found reduced proliferation of MYF progenitors during alveolarization, abnormal deposition of elastin and failure of alveolar septum formation, thereby leading to enlarged pulmonary alveoli. We identified that CPEB2 promoted PDGFRα mRNA translation in MYF progenitors and this positive regulation could be disrupted by H_2_O_2_, a hyperoxia-mimetic treatment. Moreover, decreased proliferating ability in KO MYFs due to insufficient PDGFRα expression was rescued by ectopic expression of CPEB2, suggesting an important role of CPEB2 in upregulating PDGFRα signaling for pulmonary alveologenesis.

**Conclusions:**

CPEB2-controlled translation, in part through promoting PDGFRα expression, is indispensable for lung development and function. Since defective pulmonary PDGFR signaling is a key feature of human BPD, CPEB2 may be a risk factor for BPD.

## Background

The development of the rodent lung starts from the specification and proliferation of tracheal and lung cell progenitors from the foregut endoderm at embryonic day 9.5. Subsequently, coordinated branching morphogenesis and vascular development result in a tree-like structure of epithelial tubules with differentiated airways and saccules. After birth, alveologenesis further remodels primitive saccules into mature alveoli with coordinated expansion of the microcapillary network for efficient gas exchange across the epithelial and endothelial barriers [1, 2]. Postnatal alveolar development is characterized by the expansion of epithelial cells lining primitive saccules and the formation of secondary septa by alveolar myofibroblasts (MYFs). MYFs differentiated from their progenitors migrate to the tips of growing septa and secret extracellular matrix proteins, such as collagen and tropoelastin, which provide the driving force for septal protrusion [3].

Previous studies identified that α-smooth muscle actin-positive (αSMA^+^) MYFs are derived from alveolar MYF precursor cells expressing platelet-derived growth factor receptor α (PDGFRα) [4, 5]. Activation of PDGFRα in response to epithelial cell-secreted PDGF-A is crucial for the proliferation, differentiation and migration of MYFs [6]. The PDGF signaling family of proteins includes 4 ligands (PDGF-A, B, C and D) and 2 receptors (PDGFRα and β) [6, 7]. Both PDGFRα and β are receptor tyrosine kinases with an extracellular ligand-binding motif, a transmembrane region and an intracellular tyrosine kinase domain [8, 9]. PDGF-A and -B form as a disulfide-linked homo- or hetero-dimer: PDGF-AA, PDGF-BB or PDGF-AB. PDGFRβ interacts with only B chain-containing PDGF isoforms, but PDGFRα can bind all 3 isoforms with different affinities in vitro. Ligand binding-mediated dimerization of PDGFRs triggers autophosphorylation of the tyrosine residues in their intracellular kinase domains [10, 11] and downstream signaling cascades, including the phosphoinositide 3-kinase/Akt/mammalian target of rapamycin pathway [12] and mitogen-activated protein kinase pathway [13–15].

PDGFRα signaling regulates proliferation, differentiation, migration and/or apoptosis in many cell types, so it is important for gastrulation and the development of various tissues including lung [16]. PDGFRα-knockout (KO) mice die during embryonic development, with incomplete cephalic closure and abnormal somite patterning [17]. PDGF-A and PDGFRα are essential for alveolar MYF development and pulmonary alveologenesis. PDGF-A–null mice show high lethality and lack alveolar MYF progenitors and MYFs, so surviving mice show failure to form alveolar septa and consequently emphysema-like phenotypes [18, 19]. A recent study showed that PDGF-A in type II alveolar epithelial cells (AECIIs), whose synthesis is regulated by Notch 2 signaling, secretes as a paracrine signal to activate PDGFRα of alveolar MYF progenitors. Epithelium-specific inactivation of Notch 2 results in decreased PDGF-A expression and impaired alveologenesis [20]. Similarly, neonatal rats injected with a PDGFR antagonist from postnatal day 1 (P1) to P7 showed reduced alveolar cell proliferation and defective septation to cause alveolar space enlargement with abnormal elastin deposition continuously to adulthood [21]. Thus, a transient downregulation in PDGFRα signaling during early infancy produces long-lasting changes in lung architecture and alveolar stretch ability to cause emphysematous dysfunction, reminiscent of bronchopulmonary dysplasia (BPD).

BPD is a chronic lung disease of hypoalveolarization occurring in premature infants, especially those under mechanical ventilation. PDGFRα level in lung mesenchymal stromal cells (MSCs) isolated from premature infants under ventilation was reduced in those in whom BPD later developed [22]. Similarly, neonatal mice exposed to hyperoxia showed BPD-like phenotypes with reduced development of PDGFRα-positive alveolar tips [22]. Despite the implication of defective PDGFRα signaling in BPD, much less is known about the molecular mechanism regulating PDGFRα expression during alveologenesis.

Cytoplasmic polyadenylation element-binding protein 2 (CPEB2) is a sequence-specific RNA-binding protein widely present in various tissues with high expression in the brain, liver and testes [23]. CPEB2 can bind to eukaryotic translation elongation factor 2 (eEF2) and downregulate eEF2’s GTPase activity to impede translation of hypoxia-inducible factor 1α mRNA [24, 25]. Similar to other CPEB family proteins, CPEB2 can also promote translation of some of its target mRNAs [26, 27]. Previously, we found that CPEB2-KO neonatal mice showed aberrant respiration with frequent apnea and died mostly within 3 days after birth. Although enhanced cholinergic transmission-induced bronchoconstriction accounts for some apneic episodes and possible mortality in CPEB2-KO neonates [23], inhalation of an anti-cholinergic bronchodilator only partially rescued the respiratory apnea in CPEB2-KO pups without improving survival. Thus, hyperactivated cholinergic signaling contributes to only some but not all respiratory abnormalities in CPEB2-KO mice.

In the present study, we identified that CPEB2 regulates pulmonary alveologenesis by promoting PDGFRα mRNA translation in alveolar MYF progenitors. CPEB2 deficiency renders insufficient PDGFRα signaling to impair proliferation of MYF progenitors and decrease the population and localization of MYFs at the growing tips of septa, thereby leading to enlarged pulmonary alveoli with irregular elastin deposition. Consequently, CPEB2-KO mice surviving to adulthood show pulmonary dysfunction with decreased elastance and resistance, similar to other emphysema-like mouse models and human BPD [28–30]. Moreover, we analyzed 2 GEO datasets and found decreased CPEB2 mRNA level in umbilical cords of BPD infants [31] and hyperoxia-exposed pulmonary MSCs [32]. In summary, CPEB2 plays an important role in alveologenesis and may be a potential therapeutic target for BPD.

## Methods

### Animals and anesthetization

All protocols for animal experiments were approved by Institutional Animal Care and Utilization Committee of Academia Sinica. CPEB2-WT and -KO mice were littermates from heterozygous matings, and their genotypes were determined by PCR as described previously [23]. Mice were maintained in a 12-h light, ~ 200 lx fluorescent light on at 8 am, and 12-h dark cycle with food and water ad libitum. To avoid the circadian effect on gene expression, mice were anesthetized and sacrificed at ~ 4 pm to collect lung tissues for all experiments. P0 and P3 pups were anesthetized by hypothermia, and mice of older ages were anesthetized with avertin (250 mg/kg) unless otherwise specified.

### Whole-body plethysmography

The Buxco plethysmographic system was used to record mouse respiration. Adult mice at 2–3 months old were habituated for 30 min in the animal chambers connected to the bias flow regulator, followed by 1-h recording. Breathing-induced pressure changes between the animal and reference chambers were detected by the transducer, and the barometric signals transferred to the Buxco MaxII amplifier were filtered through a bandwidth of 0–15 Hz to subtract background noises. Respiratory parameters from 1-h recording were analyzed by using FinePointe (Buxco system, DSI). To induce airway hyper-reactivity, nebulized methacholine at increasing concentrations was given and the value of Penh was calculated to determine airway resistance [33, 34].

### Analysis of airway hyper-reactivity in adult mice

Eight-week old mice were anesthetized with pentobarbital (Sigma-Aldrich, St Louis, MO) at 100 mg/kg of body weight. Anesthetized mice were tracheotomized, intubated, and mechanically ventilated at a tidal volume of 0.2 ml and a frequency of 150 breathes/min. Airway resistance and dynamic compliance in sedated mice challenging with increasing doses (1.25 to 40 mg/ml) of methacholine were measured by invasive plethysmography via the FinePointe RC system (Buxco Research Systems), according to the established protocol [35]. Changes in lung volume and tracheal pressure detected by two pressure sensors for 3 min after every dose of methacholine were calculated to derive average lung resistance, elastance and dynamic compliance.

### Analysis and counting of bronchoalveolar lavage fluid (BALF) cells

BALF was collected from tracheotomized mice by instilling 1 ml of phosphate buffered saline (PBS) containing 2% fetal calf serum (FCS) through a tracheal cannula, followed by aspiration and a second lavage of the same volume. The pooled BALF was centrifuged at 400 *xg* for 5 min. The pelleted BALF cells were resuspended in PBS/2% FCS, attached to cytospin slides by centrifugation, stained with Diff-Quik solution (Sysmex, Taiwan) and then counted.

### Plasmid construction

Mouse PDGFRα 3′-UTR was PCR-amplified from lung cDNA by using the primers 5′-CTAGTCTAGACTGACACGCTCCGGGTATC-3′ and 5′-ACGCGTCGACAAGTCATATATAATAAATCATTTATTGAAATATAAAG-3′. The amplified DNA fragment was cloned to the pGL3 promoter plasmid (Promega) by using XbaI and SalI cloning sites. The resulting plasmid was digested with XbaI and self-ligated to generate the 3′UTR 1-kb construct. The 3′UTRΔCPE construct was obtained by inverse PCR amplification by using the PDGFRα 3′-UTR plasmid and the primer pair 5′-AGCCTCTGTTTGTTGCTTCTGATGACAATCAAAGCTTGCC-3′ and 5′-GGCAAGCTTTGATTGTCATCAGAAGCAACAAACAGAGGCT-3′. Construction of the lentiviral vector expressing myc-CPEB2 was described [26].

### Luciferase reporter assay

HeLa cells (ATCC, CCL-2, have been examined without mycoplasma) were cultured in DMEM supplemented with 10% fetal bovine serum (FBS). Cells were subcultured in a 12-well plate 1 day before transfection. Each well of cells was transfected with the DNA mixture containing 0.5 μg plasmid expressing firefly luciferase reporter appended with or without PDGFRα 3′-UTR, 0.07 μg *Renilla* luciferase construct, and 0.5 μg of the plasmid expressing myc tag or myc-CPEB2 by using Lipofectamine 2000 (Invitrogen). The transfected cells were harvested the next day for dual luciferase assay (Promega) and immunoblotting.

### RNA-immunoprecipitation (RNA-IP)

A P10 mouse lung was homogenized in 2 ml lysis buffer [50 mM HEPES, pH 7.4, 150 mM NaCl, 1 mM EDTA, 0.5% Triton X-100, 0.5 mM DTT, 1X protease inhibitor mixture (Roche), and 40 U/ml RNase inhibitor (TOOLS Biotech)], incubated on ice for 30 min and then centrifuged at 12,000 *xg* for 15 min. The supernatants were equally divided and incubated with 10 μl protein G beads bound with 10 μg CPEB2 or control IgG for 3 h at 4 °C. The beads were washed 3 times with 700 μl lysis buffer to remove non-specific binding. Approximately 20% beads were used for immunoblotting and the rest were incubated in elution buffer (100 mM Tris-Cl, pH 8.0, 10 mM EDTA, 1% SDS, 20 μg/ml proteinase K) at 55 °C for 30 min, followed by phenol/chloroform extraction and isopropanol precipitation. The isolated RNAs were reverse transcribed by using an oligo-dT/random primer mixture and ImPromII Reverse Transcriptase (Promega). Quantitative PCR involved the Universal Probe Library and Lightcycler 480 system (Roche). The comparative Ct (threshold cycle value) method with the nontargeted RNA, glyceraldehyde-3-phosphate dehydrogenase (GAPDH) or β-actin mRNA as a reference, was used to calculate relative expression. The PCR primers used were PDGFRα, 5′-GCGAGTTTAATGTTTATGCCTTG-3′ and 5′-GGCACAGGTCACCACGAT-3′; PDGF-A, 5′-GATGAGGACCTGGGCTTG-3′ and 5′-GATCAACTCCCGGGGTATCT-3′: GAPDH, 5′-AAGAGGGATGCTCCCTTAC-3′ and 5′-CCATTTTGTCTACGGGACGA-3′; β-actin, 5′-CTAAGGCCAACCGTGAAAAG-3′ and 5′-ACCAGAGGCATACAGGGACA-3′.

### Primary culture of pulmonary MYFs

To culture primary MYFs, anesthetized P8–10 CPEB2 wild type (CPEB2-WT) and CPEB2-KO mice were perfused cardially with cold PBS and bronchoalveolar lavage. Isolated pulmonary tissues were washed 3 times with cold PBS and then digested in DMEM containing 1 mg/ml collagenase I, 2.5 mg/ml trypsin and 2 mg/ml DNase I at 37 °C for 30 min in 5% CO_2_ incubator. Liberated cells were filtered through sterile mesh, and pelleted at 200 *xg* for 5 min. Cell pellets were resuspended and cultured at ~ 10^5^/cm^2^ in DMEM containing 10% FBS and antibiotics. MYF cultures, in which ~ 75–80% of cells expressed αSMA, used in this study were kept under 5 passages. For western blot and RT-qPCR experiments in Fig. 6d-f and Additional file 1: Figure S6, primary MYFs were cultured and processed at the indicated time without subculture.

### In vitro wound healing assay

MYFs were plated at 10^5^/well in 12-well plates and grown for 2–3 days until > 95% confluency. Confluent cells were then starved in DMEM containing 0.5% FBS for 24 h, wounded with a pipette tip, rinsed once with 0.5% FBS DMEM, then incubated in 0.5% FBS DMEM for 24 h. Live imaging under a Leica DMI 6000B microscope was acquired for 24 h. Cell repopulation in the wounded area was analyzed at 0, 6, 12, 18 and 24 h after wounding. Cell polarity was determined by staining a Golgi-resident protein, GM130, at 6 h after scratching. Polarized cells were defined by their GM130 signal located within a 60^o^ angle perpendicular to the migration direction toward the scratched area.

### PDGF-A–induced growth assay

Cultured MYFs were infected with lentiviruses expressing GFP or myc-CPEB2 at DIV3, subcultured to coverslip-containing 12-well plate at 10^5^/well at DIV4 and then serum-starved at DIV5 for 24 h to arrest cells at G0/G1 phase, followed by the treatment of 50 ng/ml PDGF-A (PROSPEC, cat# cyt-590) for 3 h and 6 h. PDGF-A–treated MYFs were immunostained with Ki67 and αSMA antibodies to identify proliferating MYFs, which exit G0 phase and re-enter cell cycle to express Ki67 [36]. The percent of αSMA^+^ MYFs with Ki67^+^ signal was determined by using Cell Counter function in ImageJ software (National Institute of Health).

### Antibodies and chemicals

Antibodies used in the study are: αSMA (ab7817) and Ki67 (ab15580) from Abcam; GM130 (610822) from BD Biosciences; cleaved caspase-3 (9664S) and PDGFRα (3174S) from Cell Signaling Technology; BrdU (GTX26326) from GeneTex; pro-surfactant protein C (10774–1-AP) from Proteintech; caveolin-1 (sc-894), Hopx (sc-398,703) and GAPDH (sc25778) from Santa Cruz Biotechnology; β-actin (A5441) and α-tubulin (T5168) from Sigma-Aldrich; and AlexaFluor 488, 594 or 647-conjugated secondary antibodies from Invitrogen. Chemicals used in the study are: ProLong antifade (P36930) and 4′,6-diamidino-2-phenylindole (DAPI, D1306) reagents from Invitrogen; and Avertin (T48402), methacholine (A2251) and 5-bromo-2′-deoxyuridine (B9285) from Sigma-Aldrich.

### Immunoblotting

Protein extracts were prepared from equal amounts of lung or cultured cells in Laemmli sample buffer supplemented with 1X protease inhibitor cocktail (Roche) and phosphatase inhibitors (Tocris). Homogenates were boiled at 55 °C for 15 min and then centrifuged at 14,000 *xg* for 10 min to remove any insoluble debris. Protein lysates were resolved by 10% SDS-PAGE mini-gels, transferred to nitrocellulose membranes. The membranes were then blocked in 5% skim milk in 1X TBST (TBS with 0.05% Tween 20) for 60 min, followed by the incubation of primary antibodies at 4 °C overnight. Horse peroxidase-conjugated secondary antibodies were used for enhanced chemiluminescence detection (ECL-Prime, GE Healthcare Life Sciences) by Image Quant LAS 4000 (Fujifilm).

### Immunohistochemistry and image acquisition

To detect proliferating cells with thymidine analogue BrdU, mice at different ages were injected with BrdU at 12 pm (10 mg/kg) and sacrificed 4 h later to collect lung tissue. After anesthetization, the trachea was cannulated and the lung was inflated by installation of 4% formaldehyde in PBS at pressure 25 cm H_2_O for 2-month-old mice, 20 cmH_2_O for P14 and P21 mice, and 12 cmH_2_O for P10 and P7 mice for 10 min. Inflated lungs were ligated and fixed for 24 h at 4 °C before embedding in paraffin. The lung sections after dewaxing and rehydration procedures were boiled in 10 mM sodium citrate buffer pH 6 for 5 min to retrieve antigens, followed by immunofluorescence staining. All staining procedures were performed at room temperature with solutions prepared in PBS. The lung sections (5-μm thick) were permeabilized and blocked in PBS containing 10% horse serum, 3% bovine serum albumin, and 0.3% Triton X-100 for 1 h, then incubated with the designated primary antibodies overnight at 4 °C. After three washes with PBS, tissues were incubated with the corresponding Alexa Fluor-conjugated secondary antibodies for 1 h. Histological staining with hematoxylin and eosin was performed by the institutional Pathology Core staff. Sections after the removal of paraffin were stained with hematoxylin and eosin. For elastic fiber staining, lung sections after dewaxing and rehydration were rinsed with 70% ethanol and stained with aldehyde-fuchsin solution (1 g pararosanilin, 1 ml concentrated hydrochloric acid and 2 ml paraldehyde in 100 ml 70% ethanol) for 5 min. The slides were rinsed with 70% ethanol and washed with H_2_O for 3 times. Fluorescent images were acquired under a LSM780 confocal microscopy (Carl Zeiss) or Axio Imager Z1 fluorescence microscopy (Carl Zeiss).

### 3D image acquisition and reconstruction

P10 mice were injected with BrdU, anesthetized and perfused with 4% formaldehyde in PBS as described in the previous paragraph, except overnight post-fixed lungs were immersed in 20 and 30% (wt/vol) sucrose/PBS overnight and then were embedded in Tissue-Tek OCT compound. Lungs were sectioned at 60 μm by using a Leica CM1850 cryostat. Lung sections were immersed with the antigen retrieval buffer (Tris/EDTA, pH 9.0) at 95 °C for 20 min, permeabilized with 0.5% Triton X-100/PBS for 2 h and blocked in 10% horse serum and 3% BSA for 2 h at room temperature, followed by the incubation of primary antibodies in 0.5% Triton X-100/PBS at 4 °C overnight. After three washes of PBS, tissues were incubated with the corresponding Alexa Fluor-conjugated secondary antibodies at 4 °C overnight, washed with PBS for 3 times and then mounted in Antifade Mounting Medium (VECTASHIELD). Serial optical Z-sections of fluorescent images were acquired under a LSM780 confocal microscopy with a Plan-Apochromat 20x/0.8 M27 objective at the resolution of 2048 × 2048 and the total depth of 60 μm with 0.8 μm per Z-step. Three dimensional images were reconstructed by using ZEN black image analysis software (Carl Zeiss, 2011 SP7 FP3, version 14.0.0.0).

### Image quantification

Immunohistochemical and histological images acquired from 2 to 3 pulmonary sections per mouse, at least 5 mice per genotype at every postnatal age, were used for analysis. A random selection of 5 imaging fields (each field ~ 45,000 μm^2^) from 2 to 3 pulmonary sections were analyzed independently by 3 undergraduate students using cell counter and multi-measure functions in ImageJ 1.47v. Two close values from the 3 independent analyses were averaged to derive quantified results in Figs. 2b, c, 3a-d, 4c-e and 5a’-b”. For scoring the density, length, and number of elastic fibers, only signals within alveolar sacs were considered and those located in vessels, alveolar ducts, bronchioles and bronchia were omitted (an example in Additional file 1: Figure S1). For counting PDGFRα^+^ and αSMA^+^ double-positive cells, cells with both immunofluorescence signals surrounding more than a half of nucleus were counted, similarly for single positive cells (examples in Additional file 1: Figure S2). To normalize unequivalent alveolar airspace in WT and CPEB2-KO lungs, the cell density was calculated by dividing cell numbers with DAPI-positive (nuclei) area. The alveolar size was determined by morphometric lung measurements of mean linear intercept (MLI) [37]. MLI was determined by drawing 5 lines of 0.5-mm length over alveoli in each image, counting the intercepts with alveolar walls for all 5 lines and dividing 2.5 mm by the number of intercepts.

### Analysis of gene expression omnibus (GEO) datasets

To explore the expression profiles of CPEB2 and PDGFRα mRNA in BPD-related research, we used GSE8586 and GSE99633 datasets from the GEO database (http://www.ncbi.nlm.nih.gov/geo/). GSE8586 study involved using Affymetrix Human Genome U133 Plus 2.0 array to profile transcriptomes of umbilical cord tissues isolated from premature newborns in whom BPD developed or not [31]. The other GSE99633 study involved isolated CD146^+^ lung MSCs from P12 rat pups exposed to 95% or 21% O_2_ from birth to P10 [32]. The mRNA levels of designated targets were extracted.

### Data and statistical analyses

Mice and cultured cells were randomly assigned for time-course study and drug treatment, respectively. Imaging fields were randomly selected during image acquisition. The sample size among experimental groups was kept as equal as possible. GraphPad Prism 5.0 and Excel software were used to produce graphs and for statistical analysis. One-way and two-way ANOVA and two-tailed Student’s *t* test were used as appropriate for statistical analysis. Bonferroni’s multiple comparison test was used for comparisons among multiple conditions after ANOVA analysis. Sample numbers and statistical results are indicated in the figure legends. *P* < 0.05 was considered statistically significant.

## Results

### Adult CPEB2-KO mice show emphysema and alveolar enlargement

**Fig. 1:**
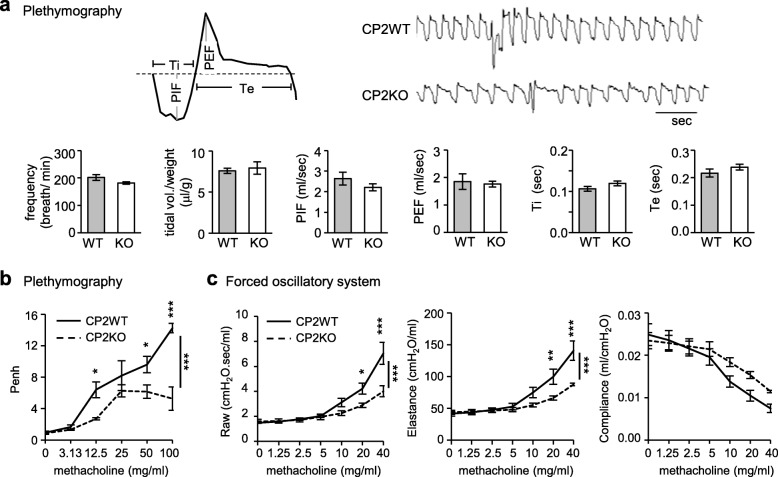
Abnormal lung function in CPEB2-KO mice. **a** Whole-body plethysmographic recording of CPEB2-WT (CP2WT) and CPEB2-KO (CP2KO) mice at age 2–3 months (*n* = 7 per group). Representative recorded traces and results of respiratory frequency, normalized tidal volume (Vt/ body weight), peak inspiratory and expiratory flow (PIF and PEF), inspiratory and expiratory time (Ti and Te). **b** Values of enhanced pause (Penh) indicating airway reactivity determined by whole body plethysmography in adult CP2WT and CP2KO mice (*n* = 4 per group) with increasing doses of inhaled methacholine (Mch). **c** Airway resistance, pulmonary elastance and dynamic compliance in adult tracheotomized mice (WT/KO = 4/5) challenged with increasing doses of Mch determined by an invasive forced oscillatory system. Data are mean ± s.e.m., *P < 0.05, **P < 0.01 and ***P < 0.001, by two-way ANOVA and Bonferroni *post-hoc* test

CPEB2-KO mice are born alive, but about two-thirds die before weaning. We previously found that loss of CPEB2 in the dorsal motor nucleus of the vagus neurons leads to translational upregulation of choline acetyltransferase, thereby increasing pulmonary acetylcholine level to induce bronchoconstriction-associated apnea in neonatal pups [23]. However, surviving CPEB2-KO mice can live for at least 1 year without obvious physical problems except for reduced thermogenesis in brown adipose tissues [27]. To investigate whether the abnormal breathing patterns in neonatal CPEB2-KO mice persist into adulthood, we used a non-invasive whole-body plethysmography (WBP) to analyze their breathing patterns. Respiratory parameters, including breathing frequency, tidal volume, peak inspiratory flow (PIF), peak expiratory flow (PEF), inspiratory time and expiratory time were comparable between CPEB2 wild-type (CP2WT) and CPEB2-KO (CP2KO) adult mice (Fig. 1a). Bronchoconstriction-associated apnea phenotypes, such as aberrant breathing pattern, reduced PIF and PEF, in CPEB2-KO neonates [23] were not observed in adults.

Unexpectedly, adult CPEB2-KO mice exhibited reduced airway resistance in response to methacholine challenges, with lower value of enhanced pause (Penh) measured by WBP in comparison with WT mice (Fig. 1b, *P* < 0.001). In addition to the non-invasive measurement, we further used an invasive forced oscillatory system to directly measure airway impedance in anesthetized CPEB2-WT and -KO adult mice. Methacholine-induced airway resistance was significantly reduced in CPEB2-KO mice (Fig. 1c, *P* < 0.001), in contrast to mice with cholinergic neuron-specific ablation of *Cpeb2* showing airway hyper-reactivity [23]. Thus, defects other than elevated cholinergic transmission should exist in global CPEB2-KO mice to cause opposite outcomes in methacholine-triggered airway response. In addition, the decrease of pulmonary elastance (Fig. 1c, *P* < 0.001) and the increased trend of dynamic compliance (Fig. 1c, *P =* 0.29) in CPEB2-KO mice indicated their compromised lung function similar to other emphysematous mice [28–30, 38, 39].

We then performed morphometric lung measurements of mean linear intercept (MLI) and found enlarged alveoli in adult CPEB2-KO lungs (Additional file 1: Figure S3a, *P* < 0.01), which is the typical morphological change in emphysematous lungs. Clinically, the marked enlargement of alveolar spaces is the hallmark of BPD and chronic obstructive pulmonary disease (COPD). However, no reduction in populations of type I AECs (AECIs) and AECIIs was observed (Additional file 1: Figure S3b). Although the number of infiltrating leukocytes in the bronchoalveolar lavage fluid (BALF) from CPEB2-KO mice were moderately increased (Additional file 1: Figure S3c, *P* < 0.01), there were no signs of inflammation because BALF cells contained only resident macrophages, and the mRNA levels of pro-inflammatory cytokines (interleukin-1β and interferon-γ) and metalloproteinase-9 were normal (Additional file 1: Figure S3d). Thus, the CPEB2-KO mouse emphysematous phenotype is unlikely caused by chronic inflammation-induced alveolar obstruction and is more reminiscent of BPD.

**Fig. 2:**
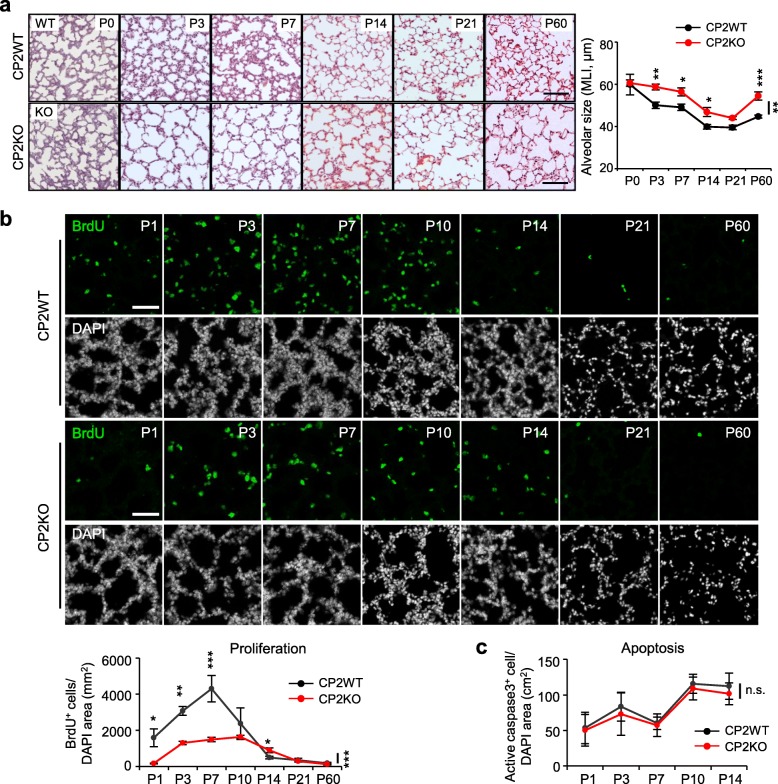
Defective alveolarization and cell proliferation in developing CPEB2-KO lungs. **a** Representative images of H&E-stained lung sections used to measure alveolar size in CP2WT and CP2KO mice at different postnatal ages (*n* = 5 mice per group at each time). MLI, mean linear intercept. Scales, 200 μm. **b** The number of BrdU-positive (BrdU^+^) proliferating cells in CP2WT and CP2KO lungs at the denoted postnatal days. Representative images of BrdU- and DAPI-labeled pulmonary sections and quantification. Scales, 50 μm. **c** Apoptosis in CP2WT and CP2KO lungs at the alveolar stage, determined by number of active caspase3^+^ cells. Data are mean ± s.e.m. (*n* = 5 mice per group at each time), n.s. not significant,*P < 0.05, **P < 0.01, and ***P < 0.001, compared with CP2WT mice by two-way ANOVA and Bonferroni *post-hoc* test

To examine whether the alveolar structure is developmentally affected by CPEB2 deficiency, we analyzed the histology and MLI of CPEB2-WT and -KO lungs at different ages. Although CPEB2-KO lungs appeared normal at birth (P0), they showed enlarged air spaces from P3 to P60 with substantial simplification of alveolar structure (Fig. 2a, *P* < 0.01), which supports that impaired lung function and altered alveolar structure are due to developmental defects during the early postnatal age.

### CPEB2 deficiency reduces cell proliferation and MYF population at the alveolar stage of lung development

Because the imbalance between alveolar cell proliferation and apoptosis during lung development accounts for emphysema-like phenotypes in many gene-ablated mouse models [39–45], we examined both scenarios in CPEB2-WT and -KO lungs at the alveolar stage [i.e., after birth to P20 in rodents [46]], to adulthood (P60) by detecting the incorporation of 5-bromo-2′-deoxy-uridine (BrdU) and active caspase-3, respectively. To normalize unequivalent alveolar airspace in WT and KO lungs, the cell density was calculated by dividing cell numbers with DAPI-positive (nuclei) area. In CPEB2-WT alveoli, BrdU-positive (BrdU^+^) proliferating cells gradually increased to peak at P7 and decreased to a barely detectable level at P21 and P60 (Fig. 2b). Although a similar proliferation curve was observed in CPEB2-KO alveoli, the number of BrdU^+^ cells was lower from P1 to P7 (Fig. 2b). Notably, proliferating cells were slightly more in CPEB2-KO lungs than WT lungs at P14. By contrast, CPEB2 depletion did not affect pulmonary apoptosis because of a similar number of active caspase 3-positive cells (Fig. 2c).

**Fig. 3:**
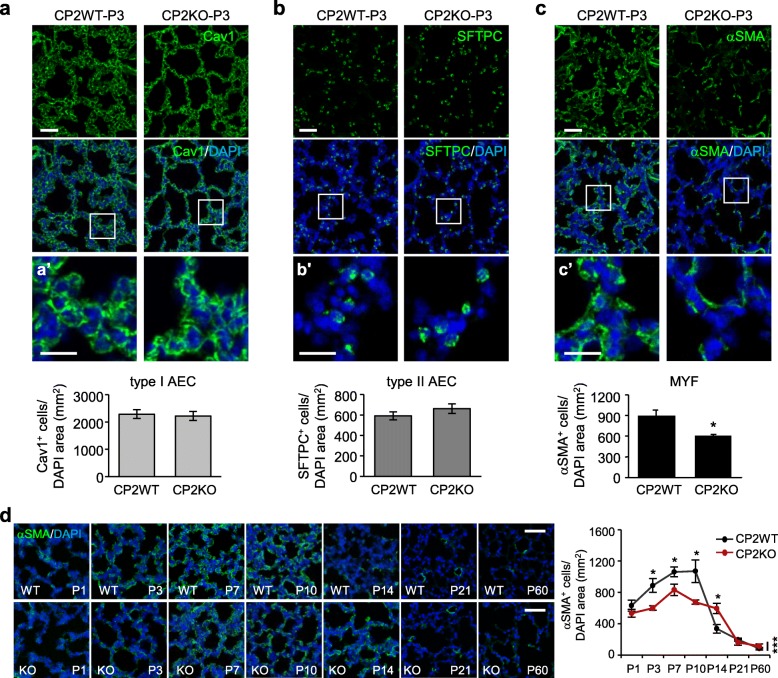
Loss of CPEB2 reduces pulmonary myofibroblasts (MYFs) during alveologenesis. **a** Caveolin 1 (Cav1^+^)-immunostained images from postnatal day 3 (P3) CP2WT and CP2KO lungs with magnified images of selected areas shown in **a’**. The number of Cav1^+^ type I alveolar epithelial cells (AECs) was quantified from 5 mice per genotype. **b,c** Similar to **a**, pro-surfactant-C–positive (SFTPC^+^) type II AECs and αSMA^+^ MYFs counted in magnified images of selected areas shown in **b’** and **c’** (5 mice per genotype). **d** The number of αSMA^+^ MYFs counted in CP2WT and CP2KO lungs (5 mice per group) at denoted ages. Data are mean ± s.e.m., *P < 0.05 and ***P < 0.001, by Student’s *t* test in **a-c** and by two-way ANOVA and Bonferroni *post-hoc* test in **d**. Scales, 50 μm in **a-d** and 25 μm in **a’-c’**

Proliferation and differentiation of alveolar cells precede alveolar septation [2]. Despite CPEB2 detected in the lung by western blot analysis [23], we could not obtain a specific CPEB2-immunostained signal in pulmonary sections after trying different antigen-retrieval and staining protocols. Nevertheless, the single cell RNA sequencing results from LungGENS [47] indicated the presence of CPEB2 mRNA in all 3 major alveolar cells, AECIs, AECIIs and MYFs, for the formation of secondary septa (Additional file 1: Figure S4a), so we first determined which alveolar cell type was mostly affected by CPEB2 depletion. AECIs line the alveoli to construct air-exchanging units and AECIIs secrete surfactants to prevent alveolar collapse during expiration [2, 20, 48]. The distribution and number of caveolin-1–positive (Cav1^+^) AECIs (Fig. 3a, a’) and pro-surfactant-C–positive (SFTPC^+^) AECIIs (Fig. 3b, b’) appeared normal in P3 CPEB2-KO lungs, which supports our observation in adult CPEB2-KO lungs (Additional file 1: Figure S3b). Moreover, the unaltered protein levels of Cav1 and SFTPC in P1 and P3 CPEB2-KO lungs (Additional file 1: Figure S4b) also reconfirmed the unaffected AECI and AECII populations in the absence of CPEB2. Due to the extended morphology of AECIs, a nuclear marker of AECIs, homeodomain-only protein x (Hopx) [49], was also examined. We observed not all Cav1^+^ AECIs expressing Hopx but the populations of Hopx–positive (Hopx^+^) AECIs in P3 WT and KO lungs were comparable (Additional file 1: Figure S4c, c’). By contrast, the number of αSMA^+^ MYFs was significantly reduced (Fig. 3c, c’, *P* < 0.05), so we further analyzed the number of αSMA^+^ cells in WT and KO lungs through the alveolar stage and adulthood. Regardless of genotype, the αSMA^+^ MYF population was transiently expanded from P1 to P10 and then vanished to almost undetectable at P21 and P60 (Fig. 3d). In CPEB2-KO lungs, the αSMA^+^ MYF population was reduced at P3, P7 and P10 but increased at P14 as compared with WT lungs.

### Mislocalized MYFs and disorganized elastic fibers in CPEB2-KO lung

**Fig. 4:**
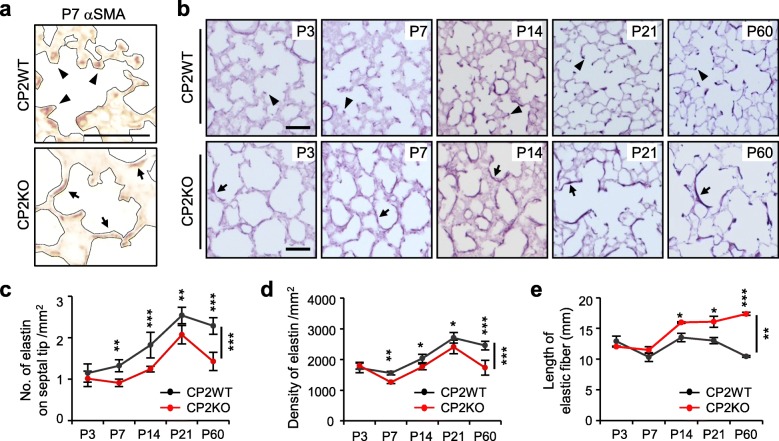
Aberrant deposition of elastic fibers in CPEB2-deficient lungs. **a** Detection of MYFs by αSMA-immunostaining in P7 CP2WT and CP2KO lungs. The alveolar structure is outlined by dashed lines. MYFs on the tips of septa and alveolar walls are denoted by arrowheads and arrows, respectively. **b** Histological morphology of elastic fibers in CP2WT and CP2KO lungs at different postnatal days. **c** The number of elastin-positive septal tips, density and length of elastic fibers in CP2WT and CP2KO alveoli (5 mice per group, representative images in **b**) at indicated postnatal ages. Data are mean ± s.e.m., *P < 0.05, **P < 0.01, and ***P < 0.001, by two-way ANOVA and Bonferroni *post-hoc* test, Scales, 100 μm

Migration of alveolar MYFs to the tips of growing septa during alveologenesis is essential for proper formation of elastin scaffold [5, 18, 19, 37]. Most αSMA^+^ MYFs already migrated to the tips of secondary septa in P7 WT lungs (Fig. 4a, arrowheads) but were trapped within the walls of enlarged alveoli in CPEB2-KO lungs (Fig. 4a, arrows). Thus, we wondered whether CPEB2-deficient MYFs showed defective migration contributing to their entrapment in alveolar walls. To measure migratory rate and polarity of MYFs, we used a wound-healing assay in cultured MYFs isolated from P10 CPEB2-WT and -KO lungs. We used immunoblotting (Additional file 1: Figure S5a) and immunofluorescence staining (Additional file 1: Figure S5b) for CPEB2 to confirm its expression in primary MYFs. Moreover, CPEB2 deficiency did not affect αSMA protein level (Additional file 1: Figure S5a), so the number of αSMA^+^ MYFs was indeed reduced in CPEB2-KO lungs (Fig. 3d). A scratch wound was inflicted on confluent MYFs and then examined for cell repopulation and orientation. When cells at the wounded edge move toward the scratched area, their Golgi organelles should position on the leading side of nuclei [50]. Although the migration rates of WT and KO MYFs were identical (Additional file 1: Figure S5c), the polarity of MYFs was significantly affected by CPEB2 depletion (Additional file 1: Figure S5d, *P* < 0.05). For approximately 65% of WT MYFs, the Golgi apparatus was oriented within a 60^o^ angle perpendicular to the migratory direction, but the percentage dropped to ~ 52% for CPEB2-KO MYFs. Hence, CPEB2 may control the intrinsic polarity of MYFs that may contribute in part to MYF migration during alveolarization.

Reduced population (Fig. 3d) and improper localization (Fig. 4a) of mature αSMA^+^ MYFs should affect alveolar elastin deposition, so we characterized the density, morphology and localization of elastic fibers in CPEB2-WT and -KO lungs at different postnatal ages (Fig. 4b). At the alveolar phase of development in WT lungs, elastic fibers were deposited along respiratory saccules and then concentrated at the tips of septa (Fig. 4b, arrowheads). By contrast, in CPEB2-KO lungs from P7 to P60, elastic fibers accumulated along alveolar walls (Fig. 4b, arrows) and were less concentrated at the tips of septa (Fig. 4c, *P* < 0.001). Although the density of elastic fibers was diminished in CPEB2-KO lungs from P7 to P60 (Fig. 4d, *P* < 0.001), the elongated fibers along septa were longer on average in CPEB2-KO lungs than WT lungs from P14 to P60 (Fig. 4e, *P* < 0.01). Together, CPEB2 deficiency resulted in reduced and mislocalized MYFs during pulmonary alveologenesis, thereby contributing to abnormal deposition of elastic fibers and emphysema-like dysfunction.

### Decreased proliferation of PDGFRα-positive progenitors in CPEB2-KO lung

**Fig. 5:**
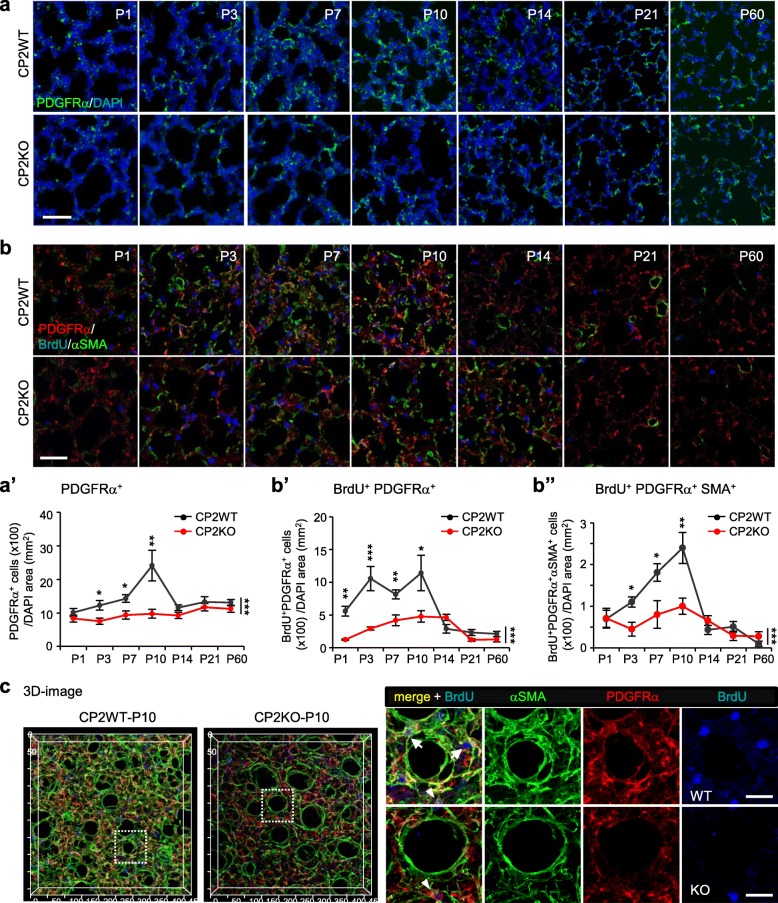
CPEB2 depletion reduces PDGFRα-expressing MYF progenitors in lungs at alveolar stages. **a,a’** Representative images and quantified results of PDGFRα-positive (PDGFRα^+^) cells in CP2WT and CP2KO lungs at denoted ages. **b** Representative images of BrdU-, PDGFRα- and αSMA-immunostained lung sections. **b’,b”** Quantification of proliferating PDGFRα^+^ (BrdU^+^PDGFRα^+^) cells and MYF progenitors (BrdU^+^PDGFRα^+^αSMA^+^) in CP2WT and CP2KO lungs (n = 5 mice per group) at indicated times. **c** 3D views of 60 μm image stacks of P10 WT and KO lungs immunostained with BrdU, PDGFRα and αSMA. Arrowheads and arrows in magnified images of selected alveoli denote BrdU^+^PDGFRα^+^ and BrdU^+^PDGFRα^+^αSMA^+^ cells, respectively. Data are mean ± s.e.m., *P < 0.05, **P < 0.01 and ***P < 0.001, by two-way ANOVA and Bonferroni *post-hoc* test. Scales, 50 μm

Because of reduced alveolar cell proliferation (Fig. 2b) and MYF population (Fig. 3d), PDGF signaling for alveologenesis may be defective in the CPEB2-KO lung to affect the proliferation of PDGFRα-expressing (PDGFRα^+^) MYF progenitors. Using MYFs cultured from lungs at different postnatal ages, we found that CPEB2 and PDGFRα levels changed in parallel. Both proteins gradually increased from P1 to P7 and decreased significantly after P14 (Additional file 1: Figure S6). Thus, we analyzed the pools of PDGFRα^+^ cells (Fig. 5a) and proliferating PDGFRα^+^ progenitors (BrdU^+^PDGFRα^+^, Fig. 5b). From P3 to P10, CPEB2 deficiency significantly decreased the number of both PDGFRα^+^ cells (Fig. 5a’, *P* < 0.001) and BrdU^+^PDGFRα^+^ progenitors (Fig. 5b’, *P* < 0.001). Because not all proliferating PDGFRα^+^ progenitors are MYF precursor cells, we also simultaneously detected αSMA. Cells containing triple positive signals (BrdU^+^PDGFRα^+^αSMA^+^) are considered MYF progenitors (magnified images and counting criteria in Additional file 1: Figure S2), a portion of proliferating PDGFRα^+^ cells committed to become MYFs, and were diminished in number in P3-P10 lungs (Fig. 5b”, *P* < 0.001). To confirm this finding not from sectioning artifacts, we used 3-dimensional reconstructed images [5] to examine P10 WT and KO lungs and observed evident reduction of BrdU^+^PDGFRα^+^αSMA^+^ cells around CPEB2-KO alveoli (Fig. 5c, Additional file 1: Figure S7, arrows).

### CPEB2 binds to and activates PDGFRα mRNA translation

**Fig. 6:**
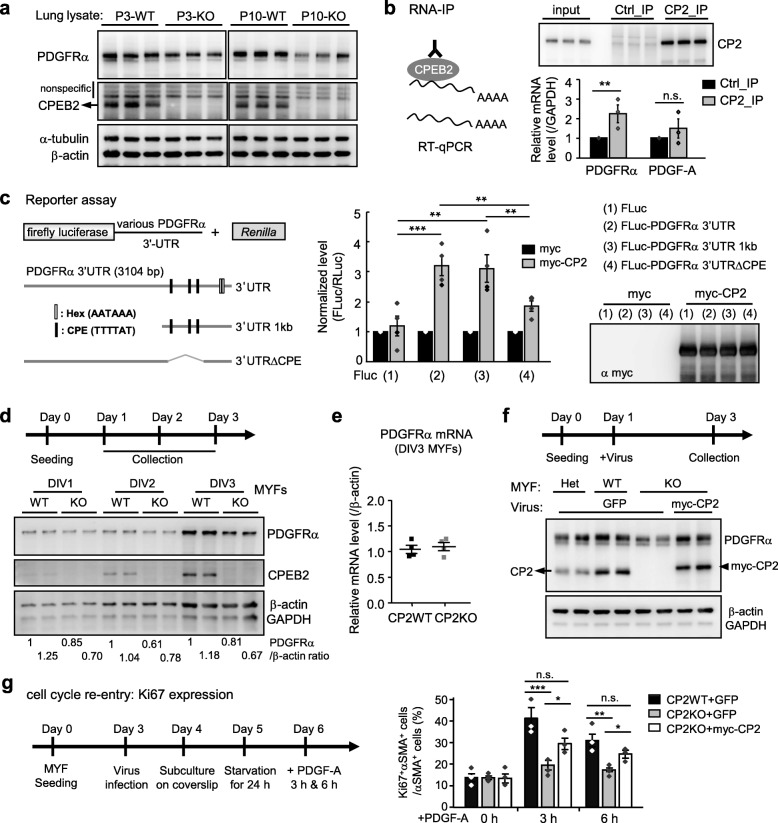
CPEB2 binds to and promotes PDGFRα mRNA translation. **a** Western blot analysis of PDGFRα protein levels in P3 and P10 lungs from CP2WT and CP2KO mice (*n* = 3 mice per group). **b** RNA immunoprecipitation (RNA-IP). P10 lung lysates were precipitated with control (Ctrl) or CPEB2 (CP2) IgG. RT-qPCR of PDGFRα and PDGF-A mRNA levels in immunoprecipitates expressed as relative ratio to the non-target control GAPDH. Data are mean ± s.e.m. from 3 independent experiments. **c** Dual luciferase reporter assay. Three CPEs and hexanucleotide (Hex) in the 3′-UTR of mouse PDGFRα mRNA are marked. The reporter plasmids, one of FLuc constructs and RLuc, were co-transfected with the plasmid expressing myc-tag or myc-CPEB2 (myc-CP2) into HeLa cells. Analysis of FLuc and RLuc activity and the expression level of myc-CP2. Data are mean ± s.e.m. from 4 independent experiments. **d** Primary MYF cultures from P10 lungs were harvested at 1, 2 and 3 days in vitro (DIV) for immunoblotting. **e** Similar to **d**, except DIV3 MYF cultures were harvested for RT-qPCR of PDGFRα mRNA level relative to GAPDH. Data are mean ± s.e.m. from 4 independent cultures. **f** Similar to **d**, except DIV1 CP2WT and CP2KO MYFs were infected with lentiviruses expressing GFP or myc-CP2 and harvested at DIV3 for immunoblotting. Het: *Cpeb2*^+/−^ MYFs. **d,f** Protein lysates were from 2 independent cultures. **g** PDGF-A–induced Ki67 expression in serum-starved MYFs. Lentivirus-infected CP2WT and CP2KO MYFs were starved in 0.5% serum for 24 h and then treated with PDGF-A for the indicated time, followed by labeling of DAPI, Ki67 and αSMA. Data are mean ± s.e.m. from 3 independent experiments. n.s. not significant, *P < 0.05, **P < 0.01 and ***P < 0.001, by Student’s *t* test in **b**, one-way ANOVA in **c** and two-way ANOVA in **g**

PDGF-A signaling is essential for lung MYF development and alveologenesis [19]. Although we tried to detect the ligand PDGF-A by using 3 different antibodies, we found no consistent staining patterns on immunohistochemistry or immunoblotting assay. In contrast, the PDGFRα signal of correct molecular weight on western blots was significantly reduced in P3 CPEB2-KO lung lysates and more evident in P10 lysates (Fig. 6a), which agrees with the immunostaining result (Fig. 5a,a’). In contrast, the PDGFRα mRNA levels in P0 and P3 lungs did not differ significantly between WT and KO groups (Additional file 1: Figure S8), so CPEB2 may regulate the PDGFRα protein level. Although the population of AECIIs remained unaltered in CPEB2-KO mice (Fig. 3b), CPEB2 may affect PDGF-A synthesis. Alternatively, CPEB2 may regulate PDGFRα synthesis in MYF progenitors to support proliferation. Both 3′-untranslated regions (UTRs) of PDGFRα and PDGF-A mRNAs contain canonical CPEB-binding elements (CPE, UUUUA_1-2_U), so we tested whether CPEB2 could associate with them in the developing lung by RNA immunoprecipitation. Using P3 lung lysates, we found that CPEB2 bound to PDGFRα but not PDGF-A mRNA (Fig. 6b). The 3′-UTR of PDGFRα mRNA contains 3 CPEs (UUUUAU) located just upstream of the poly(A) signal (AAUAAA, Fig. 6c). To investigate whether CPEB2 activates the translation of PDGFRα mRNA via its 3′-UTR, we used dual luciferase reporter assay with firefly luciferase (FLuc) appended with mouse PDGFRα 3′-UTR in HeLa cells. The *Renilla* luciferase (RLuc) plasmid was used to normalize the variation in transfection efficiency. Indeed, ectopically expressed CPEB2 upregulated the translation of FLuc reporter RNA appended with the full-length or 1-kb PDGFRα 3′-UTR, both containing 3 CPEs (Fig. 6c). However, such upregulation was abrogated when all 3 CPEs were deleted (Fig. 6c, FLuc-3′UTRΔCPE), so CPEB2 activates PDGFRα mRNA translation via binding to 3′UTR CPEs.

### CPEB2 is required for PDGFRα signaling-induced MYF proliferation

Some PDGFRα^+^ cells (Fig. 5a’), particularly those with proliferating ability (Fig. 5b’) and already expressing αSMA to become MYFs (Fig. 5b”), were reduced in number in developing CPEB2-deficient lungs, so we assessed whether the reduced PDGFRα expression was intrinsic in CPEB2-KO MYFs and MYF progenitors. We cultured primary MYFs from CPEB2-WT and -KO lungs and examined the protein and RNA levels of PDGFRα. As expected, only the protein level (Fig. 6d) but not RNA level (Fig. 6e) of PDGFRα was decreased in CPEB2-KO MYF cultures. Importantly, the amount of PDGFRα protein in CPEB2-KO MYFs could be rescued by ectopic expression of myc-CPEB2 (Fig. 6f). We further examined PDGF-A–induced cell cycle re-entry of G0/G1-arrested MYFs by Ki67-immunostaining and found that the reduced number of Ki67^+^αSMA^+^ cells in CPEB2-KO MYF culture could be rescued by ectopic expression of myc-CPEB2 (Fig. 6g and representative images in Additional File 1: Figure S9). Together, these data indicate that CPEB2 promotes translation of PDGFRα mRNA in MYF progenitors for cell-autonomous proliferation.

### Oxidative stress decreases CPEB2 level accompanied by PDGFRα downregulation

**Fig. 7:**
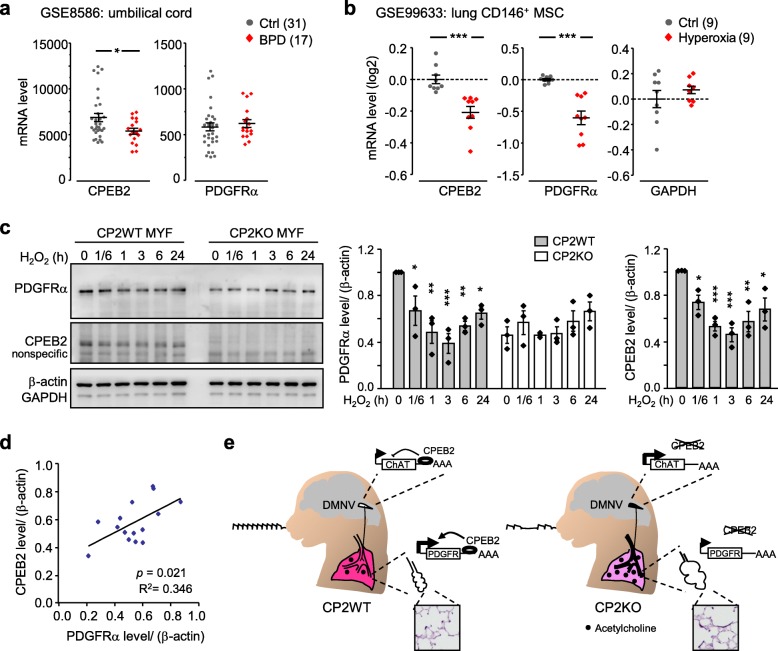
Hyperoxia-mimetic condition downregulates CPEB2 and PDGFRα expression in primary MYF culture. **a** The microarray dataset (GSE8586) containing transcriptomes of umbilical cord tissues collected from premature babies in whom bronchopulmonary dysplasia (BPD) later developed or not (Ctrl). The mRNA levels of CPEB2 and PDGFRα in these samples were retrieved. **b** The microarray dataset (GSE99633) containing transcriptomes of pulmonary CD146^+^ mesenchymal stromal cells (MSCs) isolated from neonatal rats exposed to 21% (Ctrl) or 95% oxygen (hyperoxia) for 10 days. The log2-transformed mRNA levels of CPEB2, PDGFRα and GAPDH in these samples were retrieved. **c** CP2WT and CP2KO MYF cultures were treated with 100 μM H_2_O_2_ for the indicated times and harvested for immunoblotting. PDGFRα and CPEB2 protein levels are expressed as relative ratio after normalization to β-actin level. Data are mean ± s.e.m. from 3 independent experiments. **d** Normalized CPEB2 levels in WT MYFs treated with H_2_O_2_ were correlated with PDGFRα levels in **c** by Pearson’s correlation coefficient. **e** Schematic model of CPEB2-controlled neonatal respiration. Besides CPEB2-downregulated choline acetyltransferase (ChAT) mRNA translation in dorsal motor nucleus of the vagus (DMNV) being important for normal bronchoconstriction, CPEB2-activated PDGFRα mRNA translation in MYF progenitors and MYFs is required for proper alveolarization. *P < 0.05, **P < 0.01 and ***P < 0.001, by Student’s *t* test in **a,b** and by one-way ANOVA in **c**

Loss of CPEB2 results in insufficient PDGFRα protein synthesis in MYF cultures (Fig. 6d,f), a reduced MYF population (Fig. 3d) and proliferation (Fig. 6g), and abnormal elastin deposition (Fig. 4b) for development of BPD-like phenotypes. Similarly, defective pulmonary PDGFR signaling is a key feature of human BPD and hyperoxia-treated neonatal rodents [22]. Because CPEB2-activated PDGFRα mRNA translation is important for alveologenesis, we wondered whether humans at risk of BPD may show reduced CPEB2 level. We analyzed a Gene Expression Omnibus (GEO) repository dataset (GSE8586), which contains the transcriptomic profiles of umbilical cord tissues collected from human infants born at < 28 weeks’ gestation, who later showed BPD or not [31]; the mRNA level of CPEB2 but not PDGFRα was significantly decreased in BPD infants (Fig. 7a, *P* < 0.05). In the other study (GSE99633), newborn rats under 95% hyperoxia for 10 days were sacrificed at P12 to isolate lung CD146^+^ MSCs [32]. Because these CD146^+^ MSCs also contain a PDGFRα-expressing MYF lineage during the alveolar stage of lung development [4], we analyzed this dataset and found a significant reduction in CPEB2 and PDGFRα mRNA levels in hyperoxia-exposed pulmonary MSCs (Fig. 7b, *P* < 0.001). Thus, we hypothesized that hyperoxia exposure during alveologenesis in humans and rodents may downregulate PDGFRα protein synthesis, in part by impairing CPEB2-activated translation. Hyperoxia exposure was reported to increase H_2_O_2_ production in lung and brain [51, 52], so we tested the above hypothesis by treating WT and CPEB2-KO MYF cultures with 100 μM H_2_O_2_, a hyperoxia-mimetic condition, to evaluate CPEB2 and PDGFRα expression. H_2_O_2_-reduced PDGFRα protein level was evident in only CPEB2-WT MYF culture (Fig. 7c). Although PDGFRα was downregulated in CPEB2-KO MYFs under normoxia, it was not further diminished by H_2_O_2_ (Fig. 7c). Of note, CPEB2 level was also decreased in H_2_O_2_-treated MYFs (Fig. 7c). Moreover, the protein levels of CPEB2 and PDGFRα were positively correlated after H_2_O_2_ treatment (Fig. 7d). Along with our previous finding that bronchoconstriction-associated respiratory defect is caused by elevated choline acetyltransferase mRNA translation in dorsal motor nucleus of the vagus [23], the present study identified that CPEB2 in MYFs and MYF progenitors appears to activate PDGFRα mRNA translation. Together, double-hit defects in both parasympathetic transmission and pulmonary alveologenesis affect neonatal survival in global CPEB2-KO mice (Fig. 7e).

## Discussion

Neonatal lethality in CPEB2-KO mice first revealed the regulatory role of CPEB2 in respiration. Although specific ablation of *Cpeb2* in cholinergic neurons recapitulates obstructive airway-associated apnea, the degree of severity is much reduced to cause death [23], so we initiated our investigation to search for other respiratory anomalies in global CPEB2-KO mice. Emphysema-like phenotypes in adult CPEB2-deficient mice reminded us of two hypoalveolarization diseases, BPD and COPD. BPD results from abnormal lung development, whereas COPD represents the destruction of an established alveolar structure by chronic inflammation. Because the alveolar structure changes, including alveolar size, elastic fiber deposition and secondary septum formation, take place within the 1st postnatal week in CPEB2-KO mice, the depletion of CPEB2 leads to abnormal lung manifestations reminiscent of human BPD. Lung organogenesis in both humans and mice proceeds through 5 morphogenesis phases— embryonic, pseudoglandular, canalicular, saccular and alveolar — to establish a sophisticated alveolus and microcapillary-coupled network for efficient exchange of oxygen and carbon dioxide [46]. Most BPD cases develop in preterm babies of < 28 weeks’ gestation, whose lungs are still at the saccular stage of development and are too immature to support autonomous breathing. Thus, mechanical ventilation with the amount of oxygen higher than normoxia is used to support their survival, but the pressure and excess oxygen can induce BPD manifestation in only some infants, which suggests that genetics also determines susceptibility to BPD. By contrast, CPEB2-deficiency–induced emphysematous phenotypes result from diminished PDGFRα expression in MYF progenitors to impair the alveolar phase of lung development. We found that oxidative stress could downregulate CPEB2 expression in MSCs and MYF culture (Fig. 7b and c), so defective translation together with a reduced PDGFRα mRNA level further aggravates the insufficiency of PDGFRα signaling. Therefore, the loss of CPEB2 function, due to genetic mutations or oxidative stress insult, may possibly be a risk factor for human BPD.

The critical role of PDGF/PDGFR signaling in alveolar development was first discovered in lungs of PDGF-A–null mice with reduced secondary septa and enlarged alveolar sacs [18, 19]. Specific depletion of PDGF-A in AECIIs during the alveolar phase of lung development is sufficient to impair alveolarization. PDGF-A released from AECIIs is required to support the proliferation of *Pdgfrα* promoter-driven GFP-expressing cells, including MYFs and lipofibroblasts. Moreover, this study also found increased proliferation and number of AECIIs [53]. Mice with mesenchyme-specific depletion of PDGFRα also showed impaired alveologenesis and disorganized alveolar elastic fibers [5]. Together, both studies demonstrated that PDGF-A and PDGFRα signaling from AECIIs to mesenchymal fibroblasts is necessary for alveologenesis. Because CPEB2 did not bind to PDGF-A mRNA (Fig. 6b), defective alveolar development in CPEB2-KO mice primarily resulted from reduced PDGFRα signaling in MYF progenitors (Fig. 6f,g). Notably, we found an opposite increase of proliferating cells (Fig. 2b) and αSMA^+^ MYFs (Fig. 3d) in P14 CPEB2-KO lungs, both similarly reported in PDGFR signaling-deficient rodents [5, 21]. In rats treated with the PDGFRα and β antagonist imatinib from P1 to P7, short-term blockade of PDGFR signaling induced permanent failure of alveologenesis, including reduced number of BrdU^+^ proliferating cells at P8 and P28, enlarged distal air sacs and abnormal elastin deposition [21]. Notably, a transient increase in BrdU^+^ cells in imatinib-treated lungs at P14 was reported, but such an increase was mostly contributed by vascular endothelial cells [21]. The other study used hyperoxia-exposed mice to mimic alveolar defects in human BPD and detected alveolar αSMA in hyperoxia-treated lungs at P15 when normoxia-exposed alveoli no longer contain αSMA^+^ MYFs [5], similar to what we observed in Fig. 3d. Thus, both CPEB2 deficiency and hyperoxia exposure reduce pulmonary PDGFR signaling, leading to delayed maturation of αSMA^+^ MYFs whose proliferation in WT mice has already stopped at P14.

The expression of PDGFRα is widely controlled; however, most studies focused on transcriptional regulation or mRNA stabilization. For example, Gli1 and peroxide-inducible Ets-1 have been reported to bind to the *Pdgfrα* promoter and activate *Pdgfrα* transcription [54, 55]. Tumor necrosis factor-α inhibits *Pdgfrα* transcription depending on cFos-YY1 complex formation and histone deacetylase activity [56]. Besides transcriptional control, PDGFRα mRNA level in hypoxia-exposed pulmonary artery smooth muscle cells could be downregulated by microRNA-34a (miR-34a) to impair cell proliferation and migration [57]. Interestingly, a recent study reported that miR-34a level is markedly elevated in a hyperoxia-based BPD mouse model to impair PDGFRα expression and MYF proliferation [58]. Antagonizing miR-34a function could ameliorate hyperoxia-induced hypoalveolarization [58]. Together, PDGFRα synthesis can be regulated at the posttranscriptional level in response to various oxidative stresses. In addition, another relevant study in rat pulmonary MYFs showed that interleukin 1β-induced stabilization of PDGFRα mRNA is sensitive to the protein synthesis inhibitor cycloheximide but the nature of those de novo synthesized proteins involved in stabilizing PDGFRα mRNA has not been identified [59].

The limitation of using global CPEB2-KO mice for the study is whether other pulmonary cell types may also contribute to defective alveolarization cannot be excluded. Nevertheless, the results from MYF culture study (Fig. 6f,g) clearly demonstrated that CPEB2-activated PDGFRα expression contributes to MYF proliferation, which is necessary to support alveologenesis. Because PDGFRα signaling also regulates pathological fibrosis in injured organs and tumorigenesis [10, 16, 60–62], whether CPEB2-mediated translation controls PDGFRα signaling under pathological conditions remains an open question. Moreover, analysis of the two GSE datasets suggested that decreased CPEB2 mRNA level is associated with BPD in infants (Fig. 7a) and induced by hyperoxia in pulmonary MSCs. Whether deficient CPEB2-controlled translation contributes to pulmonary diseases requires further clinical investigation. In summary, translational upregulation of PDGFRα by CPEB2 is crucial for constructing functional alveoli at the final stage of lung development to support life-long respiration.

## Conclusions

The present study identified a novel cell-autonomous mechanism by which CPEB2 upregulates PDGFRα mRNA translation to drive the proliferation of pulmonary MYFs and lung maturation. Impaired CPEB2-regulated translation during alveolar phase of lung development may be at the risk of BPD.

## Supplementary information

**Additional file 1.** Supplementary information is available online.

## Data Availability

The datasets used and/or analyzed during the current study are available from the corresponding author on reasonable request.

## Abbreviations

AECI: Type I alveolar epithelial cell
AECII: Type II alveolar epithelial cell
BALF: Bronchoalveolar lavage fluid
BPD: Bronchopulmonary dysplasia
BrdU: 5-bromo-2′-deoxy-uridine
CAV1: Caveolin-1
COPD: Chronic obstructive pulmonary disease
CPEB2: Cytoplasmic polyadenylation element-binding protein
DAPI: 4′,6-diamidino-2-phenylindole
eEF2: Eukaryotic translation elongation factor 2
FBS: Fetal bovine serum
GAPDH: Glyceraldehyde-3-phosphate dehydrogenase
Hopx: Homeodomain-only protein x
KO: Knockout
MLI: Mean linear intercept
MSC: Mesenchymal stromal cell
MYF: Myofibroblast
PDGF: Platelet-derived growth factor
PDGFRα: Platelet-derived growth factor receptor α
PEF: Peak expiratory flow
PIF: Peak inspiratory flow
SFTPC: Pro-surfactant-C
SMA: Smooth muscle actin
UTR: Untranslated region
WBP: Whole-body plethysmography
WT: Wild-type
