# Supplementary material for: CPEB2-activated PDGFRα mRNA translation contributes to myofibroblast proliferation and pulmonary alveologenesis
Source: J Biomed Sci. 2020 Apr 15;27:52. doi: 10.1186/s12929-020-00643-0 (PMC7160907; doi:10.1186/s12929-020-00643-0)
Supplement: Supplementary file 1 — Additional file 1. Supplementary information is available online. [file 12929_2020_643_MOESM1_ESM.pdf]

Additional File 1: Supplementary 9 figures

CPEB2-activated PDGFRα mRNA translation contributes to myofibroblast proliferation and pulmonary alveologenesis

Yen-Ting Lai[1,4], Hsu-Wen Chao[2,3,4], Alan Chuan-Ying Lai[1], Shu-Hui Lin[2,3], Ya-Jen Chang[1,*], and Yi-Shuian Huang[1,*]

[1]Institute of Biomedical Sciences, Academia Sinica, Taipei 11529, Taiwan
[2]Department of Physiology, School of Medicine, College of Medicine, Taipei Medical University, Taipei 11031, Taiwan.
[3]Graduate Institute of Medical Sciences, College of Medicine, Taipei Medical University, Taipei 11031, Taiwan
[4]These authors contributed equally

Only the elastic fibers in alveolar sacs (red arrows) were analyzed

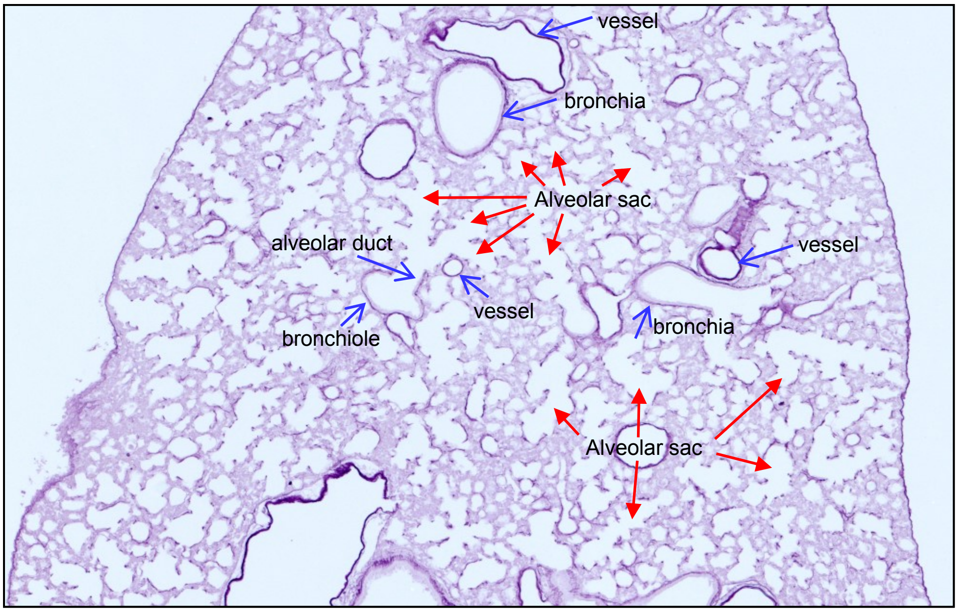

Figure S1. An example image for analyzing elastic fibers. Only alveolar elastic fibers, at the tips of secondary septa or alveolar walls, were selected for quantification in Fig. 4**c-e**.

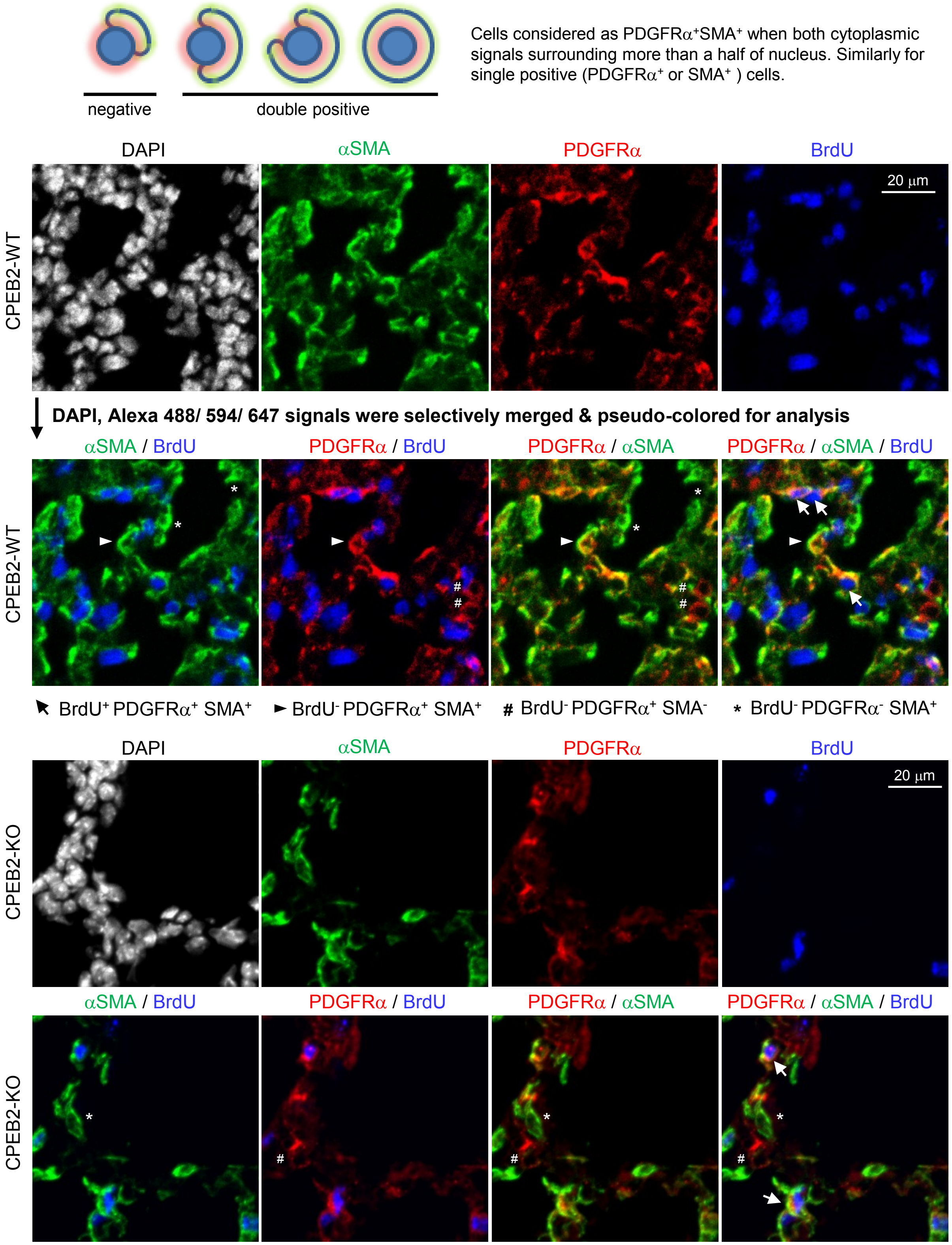

Figure S2. Example images for counting Immunofluorescence signal-positive cells. The 4-channel images taken from CPEB2-WT and CPEB2-KO pulmonary sections labelled for DAPI, αSMA, PDGFRα and BrdU could be selectively merged and pseudo-colored for image presentation and quantification.

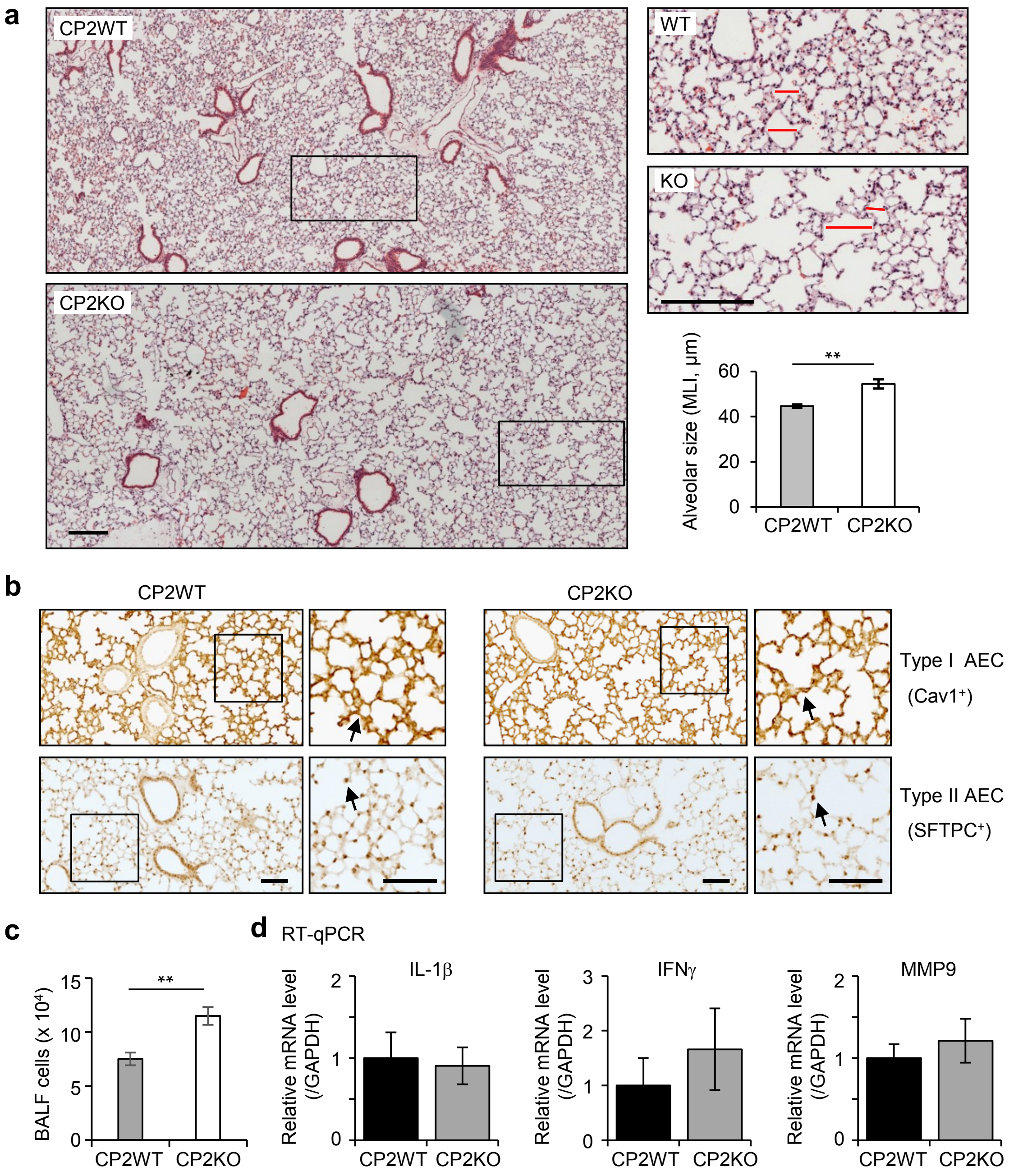

Figure S3. Enlarged alveolar space in adult CPEB2-KO mice. **a** H&E staining of lung sections prepared from adult CPEB2-WT (CP2WT) and CPEB2-KO (CP2KO) mice (P60, n = 8 mice per group). Histological analysis to determine the mean linear intercepts (MLI, examples denoted by red lines). Scales, 200 μm. **b** Immunohistochemical staining of caveolin-1 (Cav1) for type I alveolar epithelial cells (AECs) and pro-surfactant-C (SFTPC) for type II AECs in adult lung sections. Arrows denote the positive signals. Scales, 100 μm. **c** The number of leukocytes in the bronchoalveolar lavage fluid (BALF) from CP2WT (n = 5) and CP2KO mice (n = 6). **d** Lungs were isolated from adult CP2WT and CP2KO mice (n = 4-5 per group) for RT-qPCR of IL-1β, IFNγ and MMP9 mRNA levels relative to GAPDH. Data are mean ± s.e.m, $^{**}P < 0.01$, by two-tailed Student's *t* test.

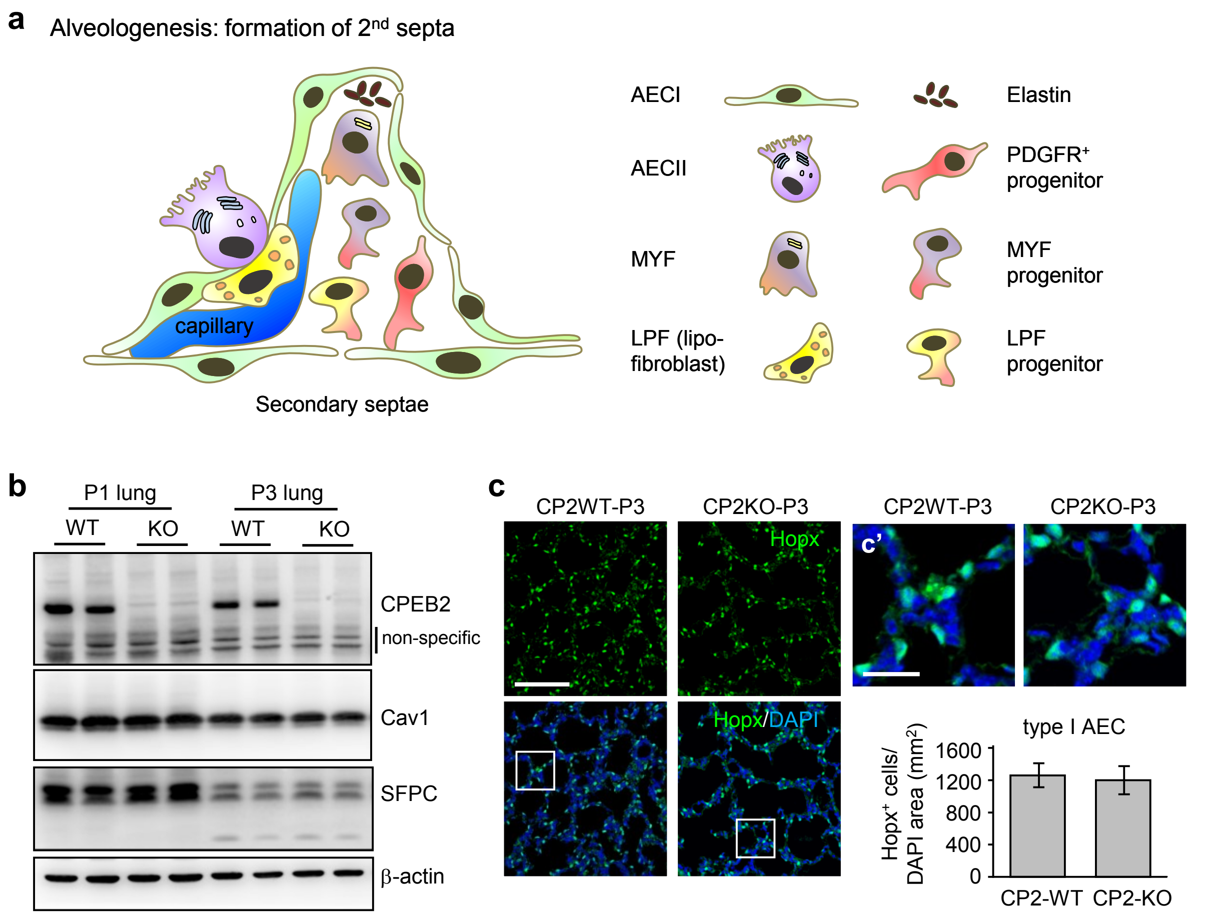

Figure S4. CPEB2 deficiency does not affect the populations of type I and type II AECs. **a** Illustration of alveolar cells involved in the formation of secondary septa during the alveolar phase of lung development. **b** Immunoblotting of caveolin-1 (Cav1) for type I AECs and pro-surfactant-C (SFPC) for type II AECs in P1 and P3 lung lysates. **c** Hopx-immunostained images from P3 WT and CPEB2-KO lungs with magnified images of selected areas shown in **c'**. The number of Hopx+ type I AECs was quantified from 5 mice per genotype. Data are mean ± s.e.m. Scales, 100 μm in **c** and 25 μm in **c'**.

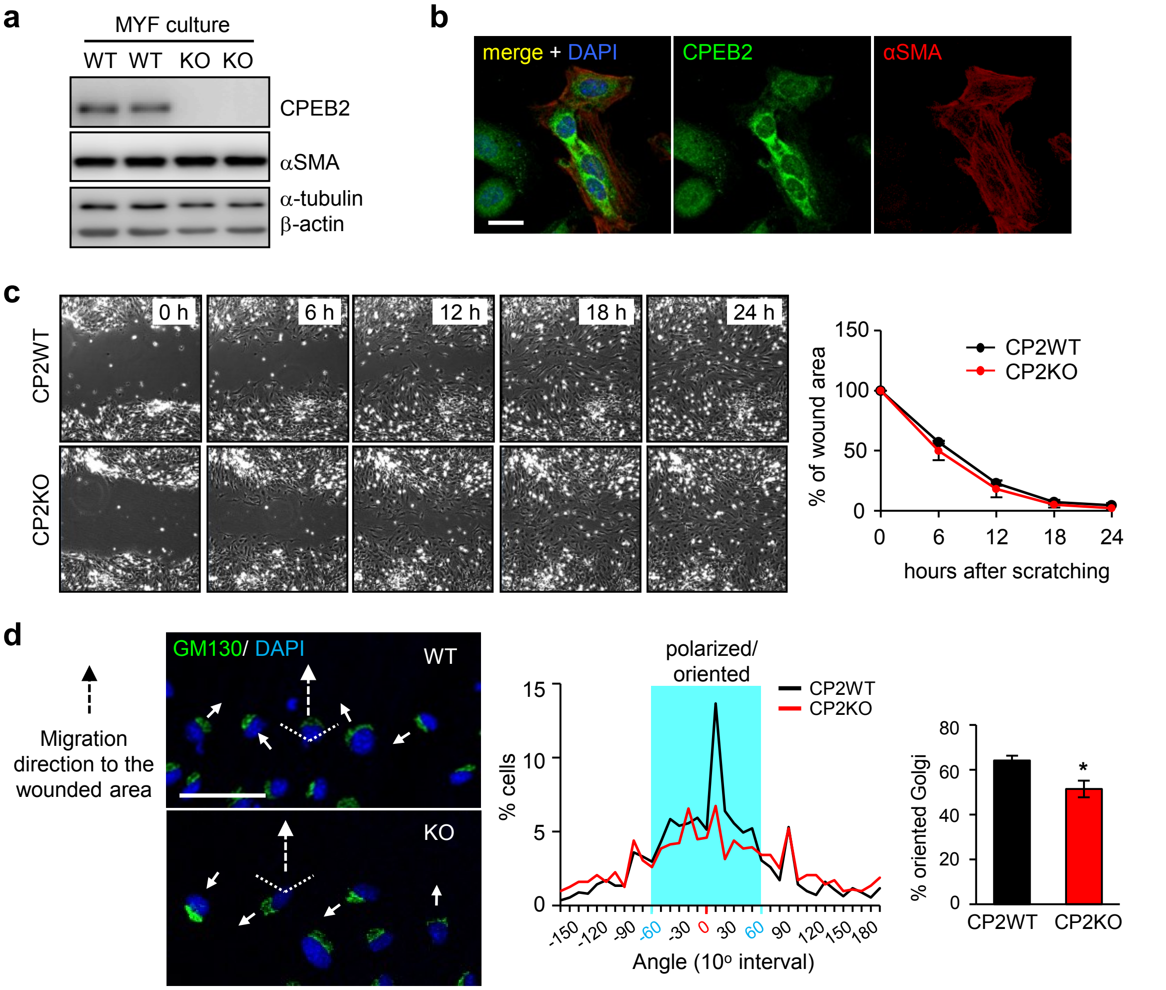

Figure S5. CPEB2 deficiency impairs polarity of MYFs during migration. **a** Primary pulmonary MYF cultures were harvested for immunoblotting of CPEB2, αSMA, α-tubulin and β-actin. **b** Cultured pulmonary MYFs were fixed for immunostaining of CPEB2 and αSMA and DAPI-labeling. Scale, 20 μm. **c** Time-lapse images and quantification of WT and CPEB2-KO MYF repopulation in wound-healing assay. The wounded areas at the denoted time after scratching were measured, with the area at 0 h set to 100%. Data are mean ± s.e.m from 4 independent experiments. **d** Cell/Golgi polarity assay. CPEB2-WT and -KO MYF cultures at 6 h after scratching were fixed for DAPI-labeling and immunostaining of the Golgi marker GM130. Polarized (or oriented) MYFs were defined when the GM130-stained Golgi was oriented within a 60° angle perpendicular to the migration direction toward the wound. Data are mean ± s.e.m. from 3 independent experiments, *$P < 0.05$, by two-tailed Student's *t* test. Scale, 100 μm.

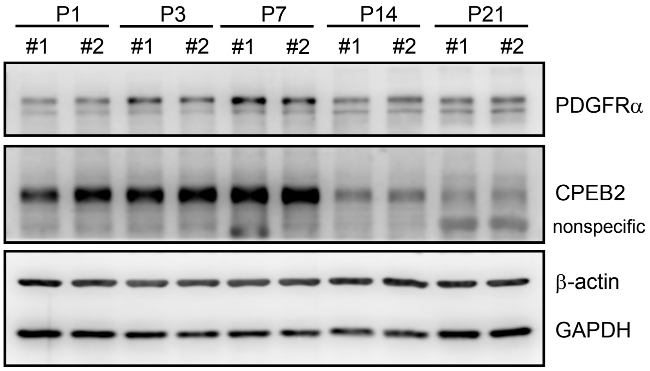

Figure S6. The expression of CPEB2 and PDGFRα in MYFs isolated from different postnatal ages. Primary MYFs were cultured from P1, P3, P7, P14 and P21 lung tissues (2 independent cultures from 2 mice per postnatal day) and then harvested at DIV1 for immunoblotting of PDGFRα, CPEB2, β-actin and GAPDH.

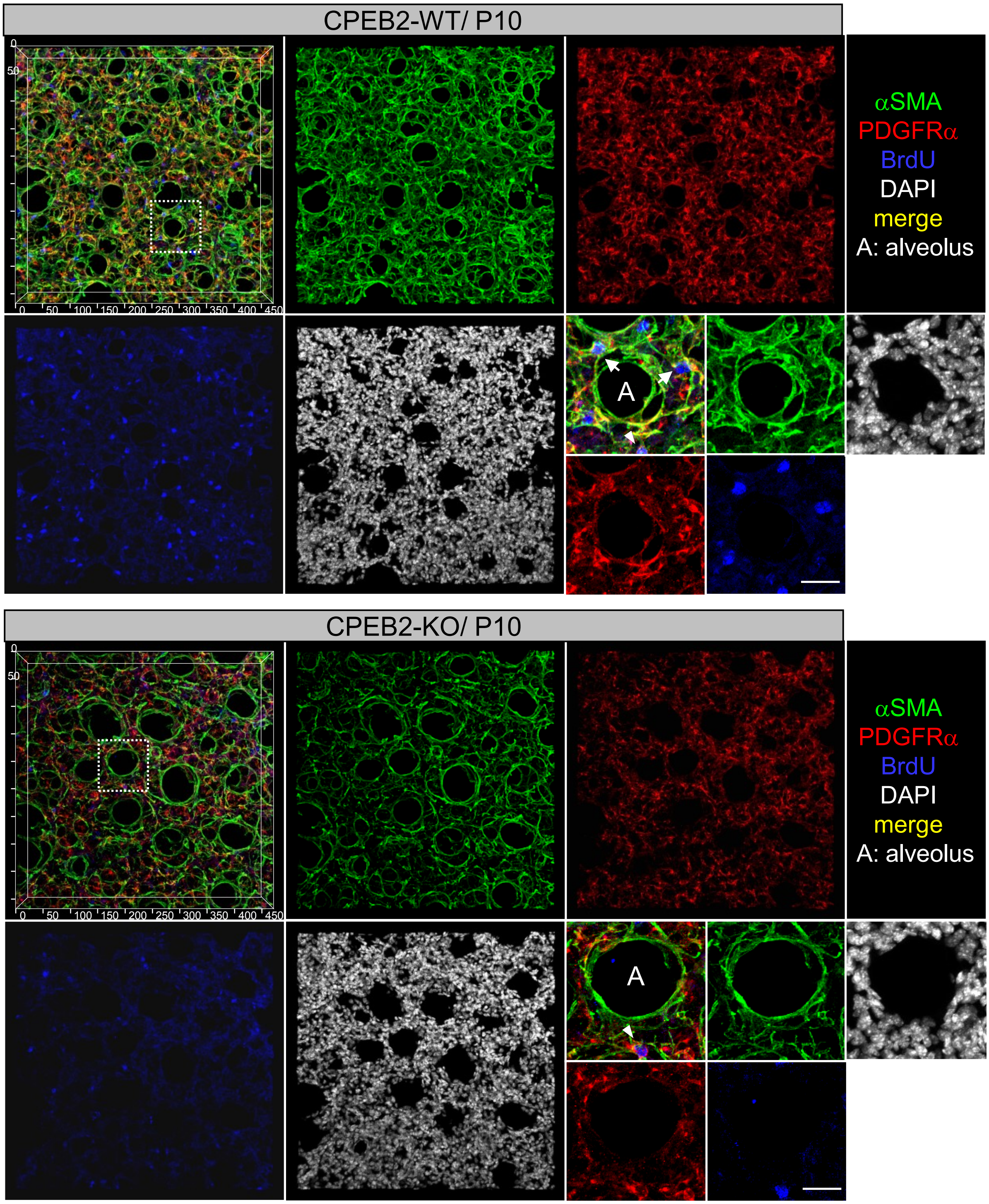

Figure S7. Reduced proliferating MYF precursors in CPEB2-KO lung. 3D rendering images of P10 WT and KO lungs immunostained with BrdU, PDGFRα and αSMA. Arrowheads and arrows in magnified images of selected alveoli denote $BrdU^{+}PDGFR\alpha^{+}$ and $BrdU^{+}PDGFR\alpha^{+}\alpha SMA^{+}$ cells, respectively. Scales, 50 μm.

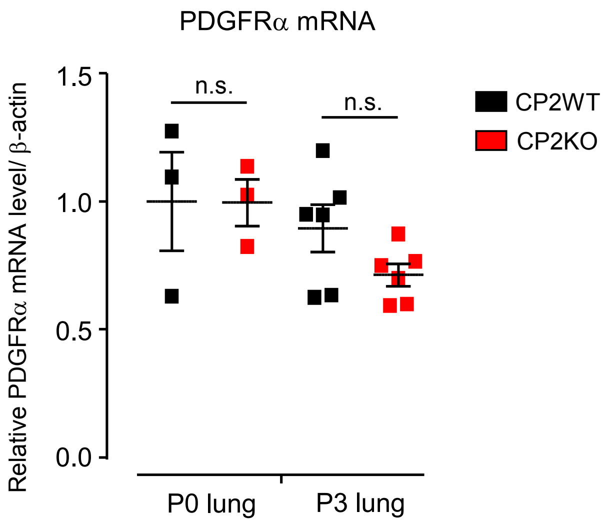

Figure S8. Unaltered PDGFRα mRNA level in CPEB2-KO lung. P0 and P3 CP2WT and CP2KO lungs were harvested for RT-qPCR of PDGFRα mRNA level relative to β-actin. Data are mean ± s.e.m. from 3 mice per genotype at P0 and 6 mice per genotype at P3.

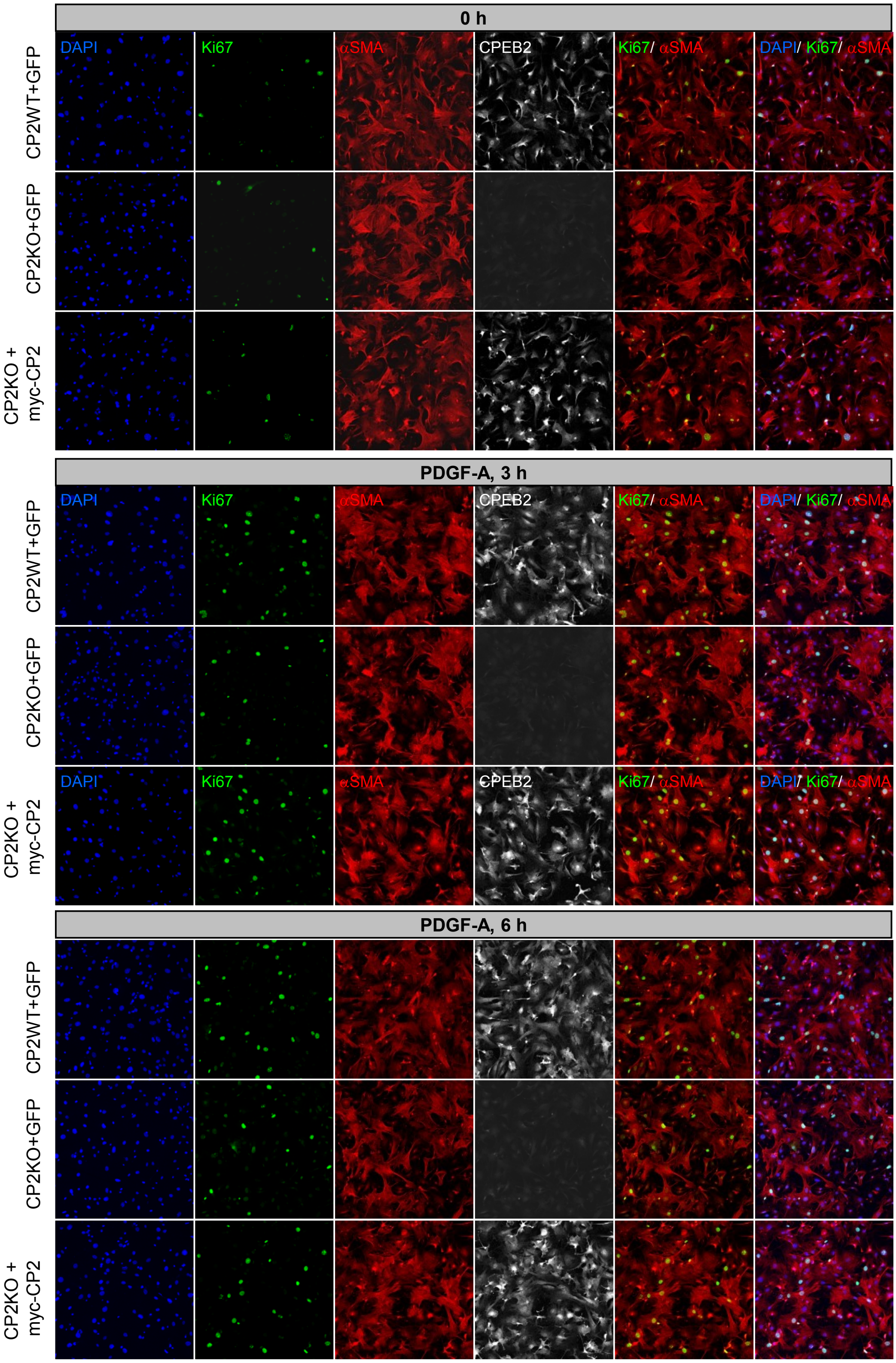

Figure S9. Impaired PDGF-A–induced Ki67 expression in CPEB2-KO MYF culture. CPEB2-WT and CPEB2-KO MYFs infected with lentiviruses expressing GFP or myc-CPEB2 were treated with PDGF-A for the indicated time, followed by labeling of DAPI, CPEB2, Ki67 and αSMA. Representative images of each time point are shown. Because of low GFP level, its auto-fluorescence did not interfere with Ki-67-immunostained signal.
